# Design Characteristics Influence Performance of Clinical Prediction Rules in Validation: A Meta-Epidemiological Study

**DOI:** 10.1371/journal.pone.0145779

**Published:** 2016-01-05

**Authors:** Jong-Wook Ban, José Ignacio Emparanza, Iratxe Urreta, Amanda Burls

**Affiliations:** 1 Evidence-Based Health Care Programme, Department of Continuing Education, Kellogg College, University of Oxford, Oxford, United Kingdom; 2 CASPe, CIBER-ESP, Clinical Epidemiology Unit, Hospital Universitario Donostia, San Sebastian, Spain; 3 School of Health Sciences, City University London, London, United Kingdom; University Medical Center Rotterdam, NETHERLANDS

## Abstract

**Background:**

Many new clinical prediction rules are derived and validated. But the design and reporting quality of clinical prediction research has been less than optimal. We aimed to assess whether design characteristics of validation studies were associated with the overestimation of clinical prediction rules’ performance. We also aimed to evaluate whether validation studies clearly reported important methodological characteristics.

**Methods:**

Electronic databases were searched for systematic reviews of clinical prediction rule studies published between 2006 and 2010. Data were extracted from the eligible validation studies included in the systematic reviews. A meta-analytic meta-epidemiological approach was used to assess the influence of design characteristics on predictive performance. From each validation study, it was assessed whether 7 design and 7 reporting characteristics were properly described.

**Results:**

A total of 287 validation studies of clinical prediction rule were collected from 15 systematic reviews (31 meta-analyses). Validation studies using case-control design produced a summary diagnostic odds ratio (DOR) 2.2 times (95% CI: 1.2–4.3) larger than validation studies using cohort design and unclear design. When differential verification was used, the summary DOR was overestimated by twofold (95% CI: 1.2 -3.1) compared to complete, partial and unclear verification. The summary RDOR of validation studies with inadequate sample size was 1.9 (95% CI: 1.2 -3.1) compared to studies with adequate sample size. Study site, reliability, and clinical prediction rule was adequately described in 10.1%, 9.4%, and 7.0% of validation studies respectively.

**Conclusion:**

Validation studies with design shortcomings may overestimate the performance of clinical prediction rules. The quality of reporting among studies validating clinical prediction rules needs to be improved.

## Introduction

Clinical prediction rules help clinicians address uncertainties surrounding the diagnosis, prognosis or response to treatment using information from individual patient’s history, physical examination and test results [1–3]. Contrary to the traditional approach where intuition is typically used to handle clinical uncertainties, clinical prediction rules enable clinicians to explicitly integrate information from individual patients and estimate the probability of an outcome.

Once a clinical prediction rule is constructed in a derivation study by combining variables predictive of an outcome, the reproducibility and generalizability of the clinical prediction rule should be evaluated in validation studies [4–8]. A clinical prediction rule that performed well in a derivation may not fare so well when it is applied to different populations or settings [6, 9–11]. Therefore, only clinical prediction rules that have proven performance through external validations should be trusted and considered for application in clinical practice [5, 12].

There have been several methodological standards proposed over the past three decades that considered design and reporting characteristics of studies deriving, validating as well as assessing the impact of clinical prediction rules [1–3, 5, 8, 12, 13]. Despite the available methodological standards, overall methodological qualities of clinical prediction rule research described in previous reports have been far from optimal [1, 2, 14–17]. However, the findings of these reports were largely based on the evaluation of derivation studies while including a limited number of validation studies. Recently, a systematic review of multivariable prediction models collected from core clinical journals showed that important methodological characteristics are poorly described in validation studies [18].

There is a growing body of empirical evidence showing that the design and conduct of a study can influence the results. For example, a number of meta-epidemiological studies which examined clinical trials included in meta-analyses have shown that failure to ensure proper random sequence generation, allocation concealment or blinding can lead to the overestimation of treatment effects [19–22]. In diagnostic test accuracy studies, it has been suggested that the use of less than optimal study design characteristics such as retrospective data collection, nonconsecutive subject selection or case-control design may lead to overestimated test accuracy [23, 24]. For validation studies of clinical prediction rules, the potential implications of employing design characteristics that are not compatible with currently available methodological standards are yet to be determined.

Our primary objective was to evaluate whether validation studies conducted using design characteristics that are inconsistent with methodological standards are associated with the overestimation of predictive performance. We also aimed to estimate the proportion of published validation studies that clearly reported important methodological characteristics so that the readers could assess the validity.

## Materials and Methods

### Reporting and design characteristics of studies validating clinical prediction rule

The methodological standards for clinical prediction rules [1–3, 13] as well as quality assessment tools and a reporting guideline for diagnostic test accuracy studies [25–27] were reviewed to identify reporting and design characteristics of studies validating clinical prediction rules. Definitions of 7 reporting characteristics and 7 design characteristics examined in our study are outlined in Table 1.

**Table 1:** Definitions of (a) reporting and (b) design characteristics.

**(a) Reporting characteristic**	**Definition**	
Population	Age, sex and important clinical characteristics are described	
Study site	Geographic location, institution type and setting, and how patients were referred are described	
Prediction rule	Clear and detailed descriptions of predictor variables and prediction rule are provided with a description of process to ensure accurate assessment of rule such as training	
Reliability	Intra-observer or inter-observer reliability of prediction rule is described	
Outcome	Clear and detailed definition of outcome is provided	
Results	Estimates of predictive performance are presented with confidence intervals	
Follow up	Clearly describes what happened to all enrolled patients	
**(b) Design characteristic**	**Level**	**Definition**
Sample size	Adequate	At least 100 patients with outcome and 100 patients without outcome
	Inadequate	Less than 100 patients with outcome or 100 patients without outcome
Patient selection	Consecutive	All consecutive patients meeting inclusion criteria are selected
	Nonconsecutive	Selection methods other than consecutive selection were used
Disease spectrum	Cohort	Prediction is ascertained before outcome is determined
	Case-control	Prediction is ascertained after outcome is determined
Validation type	Broad	Validation in different settings, with different patients and by different clinicians
	Narrow	Validation in similar settings, with similar patients or by similar clinicians
	Internal	Validation using methods such as split-sample validation, cross-validation or bootstrap
Assessment	Blind	Assessment of prediction without the knowledge of outcome and assessment of outcome without the knowledge of prediction
	Non-blind	Assessment of prediction with the knowledge of outcome or assessment of outcome with the knowledge of prediction
Verification	Complete	All predictions are verified using the same reference standard
	Partial	Only a subset of predictions are verified
	Differential	A subset of predictions are verified using different reference standard
Data collection	Prospective	Data collection for validation study is planned before prediction and outcome are assessed
	Retrospective	Prediction and outcome are assessed before data collection for validation starts

Simulations have shown that validation studies with less than 100 patients with and without an outcome may not identify the invalidity of a regression model [11, 28]. Case-control design, nonconsecutive enrollment, and retrospective data collection may lead to a biased selection of patients [29, 30]. Case-control design was associated with the overestimation of diagnostic test accuracy in a meta-epidemiological study [23]. Case-control design may be obvious when patients with clinical suspicion and healthy subjects without clinical suspicion are recruited separately [31]. However, it may be indistinct when an outcome is determined before the prediction is assessed in a “reversed-flow" design [31]. Knowing the result of the prediction may influence the assessment of an outcome and knowing the outcome of a patient may influence estimating the prediction [1–3, 13] although blinding did not significantly influence diagnostic test accuracy in meta-epidemiological studies [23, 24]. Partial or differential verification of predictions may lead to verification bias. Differential verification was associated with the overestimation of diagnostic accuracy [23, 24]. Even though validation type is not a methodological standard, this feature was included in our meta-epidemiological analysis since the type of validation is likely to influence the performance of a clinical prediction rule in validation.

### Selection of systematic reviews and validation studies

Medline, EMBASE, the Cochrane library and the Medion database (www.mediondatabase.nl) were searched for systematic reviews of clinical prediction rule studies. Searches were limited to the systematic reviews published between 2006 and 2010. No language restriction was applied. All electronic data base searches were conducted on 11 November 2011.

The search strategies for electronic databases were constructed by combining terms from validated filters for clinical prediction rule study and systematic review [32–35]. Search strategies for Medline (OvidSP™, 1946 to 2011), EMBASE (OvidSP™, 1974 to 2011), the Cochrane library, and the Medion database (www.mediondatabase.nl) are presented in S1 Fig.

Selection of studies was carried out in two steps. First, eligible systematic reviews of clinical prediction rule studies were identified from the results of the electronic database search. Then, eligible validation studies of clinical prediction rule included in these systematic reviews were selected for data extraction.

A systematic review of clinical prediction rule studies was eligible if:

1. it examined a clinical prediction rule which is defined for this study as a tool that produces a probability of an outcome to help clinicians with the diagnosis, prognosis or treatment decision by combining three or more predictor variables from patient’s history, physical examination or test results,
2. it meta-analyzed the results of clinical prediction rule studies,
3. at least one meta-analysis pooled the results of four or more validation studies of a same clinical prediction rule,
4. a diagnostic 2 by 2 table could be constructed from four or more validation studies, and
5. the primary studies were not selected or excluded based on any of the seven study design characteristics examined in this study.

Once a systematic review of clinical prediction rule studies was determined eligible, full text articles of all clinical prediction rule studies included in the systematic review were reviewed. A clinical prediction rule study was included if:

1. it was a validation study of clinical prediction rule in which the generalizability of an existing clinical prediction rule was assessed by comparing its prediction with a pre-specified outcome, and
2. the information from the study allowed for the construction of a diagnostic 2 by 2 table.

### Data extraction and analysis

From each eligible validation study, a diagnostic 2 by 2 table was constructed and the reporting and design characteristics were recorded. A systematic review may contain multiple meta-analyses that pooled the results of clinical prediction rule studies using various thresholds. Therefore, the following strategy was used to identify an optimal threshold for data extraction from a validation study. The strategy was aimed at including the maximum number of validation studies while minimizing heterogeneity of data due to threshold effects and preventing a validation study from being included in multiple analyses.

1. when the results of clinical prediction rule studies were meta-analyzed at a single threshold, this threshold was used for our data analysis.
2. when the results of clinical prediction rule studies were meta-analyzed at multiple common thresholds, the threshold that would maximize the number of eligible validation studies was chosen for data extraction.
3. when the results of clinical prediction rule studies with various thresholds were meta-analyzed and the thresholds of validation studies used in meta-analysis are known, each threshold that was chosen by authors of the systematic review to conduct meta-analysis was used.

Meta-epidemiological studies evaluate biases related to study characteristics by collecting and analyzing many meta-analyses. A multilevel logistic regression is commonly used in meta-epidemiological studies but it may underestimate standard errors and is mathematically complex since an indicator variable is required for each primary study and each meta-analysis [36]. We used a “meta-meta-analytic approach” to examine the influence of design characteristics on the performance of clinical prediction rule which involves two steps, a meta-regression and a meta-analysis of regression coefficients [36].

Firstly, a multivariable meta-regression was conducted in each meta-analysis for seven design characteristics using Meta-Disc [37] which carries out a meta-regression by adding covariates to the Moses-Littenberg model [38, 39]. The output of the meta-regression in Meta-Disc includes a coefficient which is the natural logarithm of relative diagnostic odds ratio (RDOR) and a standard error of coefficient. Performance of a clinical prediction rule in a validation study can be presented with a diagnostic odds ratio (DOR). A relative diagnostic odds ratio (RDOR) is a ratio of the DOR of a prediction rule with a certain study characteristic (e.g. case-control design) and the DOR of a prediction rule without the study characteristic. In other words, a RDOR represents the influence of a study characteristic on the performance of clinical prediction rules. As a significant amount of heterogeneity was expected between validation studies of a clinical prediction rule, meta-regressions were conducted under the random-effects assumption.

Secondly, the results of meta-regressions were meta-analyzed using Stata 12 [40]. The regression coefficients computed in the first step were meta-analyzed using the DerSimonian and Laird option in Metaan command [41]. Then, the summary RDORs were obtained from the antilogarithms of the meta-analyses results. A significant heterogeneity was assumed to be present between the results of meta-regressions and random-effects meta-analyses were conducted.

The search, selection, data extraction were completed by one of the authors (J.B.). Two clinical epidemiologists (J.E. and I.U.) independently extracted a second set of data from a random sample of validation studies included in data analysis for comparison. The agreements on the assessments of six design characteristics were evaluated using Cohen’s kappa.

## Results

### Selection of material and description of sample

The selection processes for eligible systematic reviews and validation studies are illustrated in S2 Fig. From 46848 references found in the electronic database search, 15 eligible systematic reviews consisting of 31 meta-analyses were identified [42–56]. There were 287 validation studies from 229 clinical prediction rule articles included in these meta-analyses that met the inclusion criteria.

The description of the 15 systematic reviews included in the study are outlined in S1 Table. Although the systematic reviews of clinical prediction rule published between 2006 and 2010 were searched, 13 of 15 systematic reviews in the sample were from either year 2009 or 2010. Eleven systematic reviews evaluated diagnostic prediction rules whereas four systematic reviews examined prognostic prediction rules. Meta-analyses generally contained a small number of validation studies. Twenty one meta-analyses (67.7%) included 10 or less validation studies. Only one (3.2%) meta-analysis was conducted with more than twenty validation studies. Fifteen meta-analyses evaluated objective outcomes such as the presence of ovarian malignancy [46] whereas sixteen meta-analyses evaluated subjective outcomes such as the diagnosis of postpartum depression [47].

Two hundred and seventy eight validation studies (96.9%) were written in English. There were only 12 validation studies conducted before 1991. The number of validation studies increased to 110 between 1991 and 2000 and to 165 between 2001 and 2010.

### Quality of reporting in validation studies of clinical prediction rule

**Fig 1:**
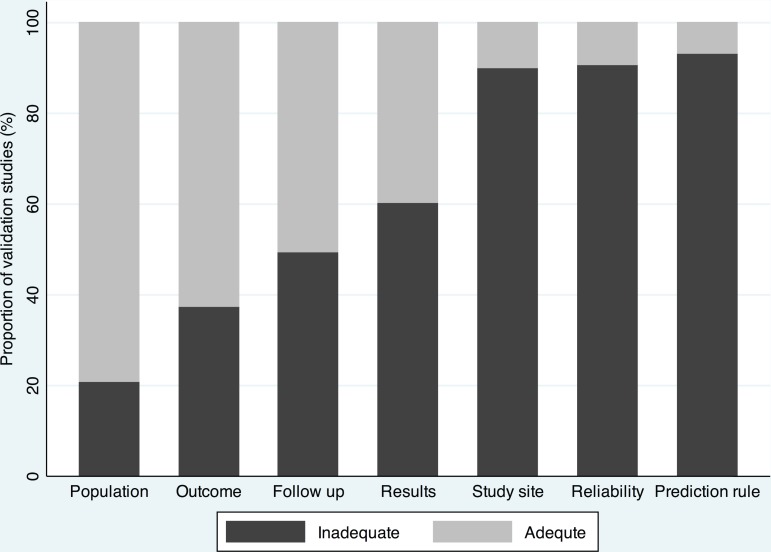
Quality of reporting. Proportion of validation studies with adequate and inadequate description of reporting characteristics.

As presented in Fig 1, validation studies providing insufficient descriptions of reporting characteristics were common. Study site, reliability, and clinical prediction rule were inadequately reported in the majority of validation studies. For reporting of study site, 118 (41.1%), 113 (39.3%) and 241 (84.0%) validation studies provided insufficient descriptions of location, institution type and referral to study site respectively. For reporting of clinical prediction rule, the processes to ensure an accurate application of prediction rule such as training were described in 20 (7.0%) validation studies. Predictive performance was reported with confidence interval in 84 (36.7%) validation studies. Although 145 (50.5%) validation studies provided information on the follow up of patients, flow diagram was used only in 23 (8.0%) validation studies.

**Fig 2:**
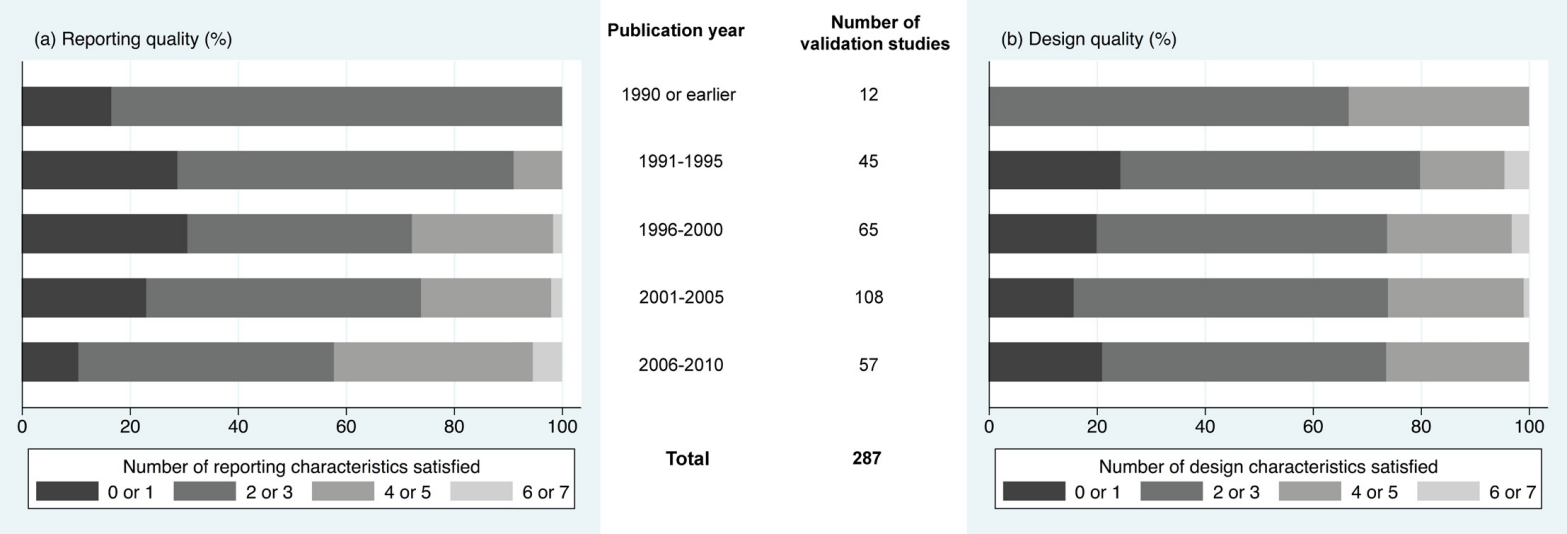
Evolution of methodological quality over time. Proportion of validation studies satisfying (a) reporting characteristics and (b) design characteristics recommended in methodological standards.

There were 66 (23%) validation studies meeting 0 or 1 reporting characteristic and 147 (51.2%) validation studies satisfying 2 or 3 reporting characteristics. Although only 74 (25.8%) validation studies met 4 or more reporting characteristics, the proportion of validation studies satisfying 4 or more reporting characteristics increased from 8.9% between 1991 and 1995, to 27.7% between 1996 and 2000, to 25.9% between 2001 and 2005, and to 42.1% between 2006 and 2010 as seen in Fig 2(A).

### Influence of design characteristics on predictive performance of clinical prediction rule

Design shortcomings and insufficient descriptions of design characteristics were prevalent among validation studies of clinical prediction rule as presented in S2 Table.

There were 53 (18.5%) validation studies meeting 0 or 1 design characteristic and 161 (56.1%) validation studies satisfying 2 or 3 design characteristics. There were 73 (25.4%) validation studies conducted while satisfying 4 or more design characteristics. The proportion of validation studies satisfying 4 or more reporting characteristics did not improve over time as seen in Fig 2(B). Between 2006 and 2010, there were 15 (26.3%) validation studies that satisfied 4 or more design characteristics.

Fisher’s exact test showed that patient selection (*p* = 0.028), disease spectrum (*p* = 0.008), and data collection (*p* = 0.00) significantly varied between validation studies of diagnostic and prognostic prediction rules. For example, nonconsecutive selection was more common among diagnostic rules (17.5%) compared to prognostic rules (4.8%). Case-control design was also more prevalent among diagnostic rules (21.6%) compared to prognostic rules (4.8%). On the other hand, retrospective data collection was more frequently found in prognostic rules (50%) than diagnostic rules (16.7%). Although there was no significant overall variation in verification between diagnostic and prognostic rules, all studies using differential verification validated diagnostic prediction rules.

Validation studies using inadequate sample size and differential verification were associated with the overestimation of predictive performance in univariable analysis as presented in S2 Table. In order to construct a multivariable model, some of the levels for design characteristics were collapsed according to the summary RDORs from the univariable analysis. Consecutive selection and unclear patient selection were collapsed and compared with non-consecutive selection. Case-control design was compared after cohort design and unclear spectrum were combined. Narrow validation was compared with a combined category of broad validation and unclear validation type. Differential verification was compared with a combined category of complete, partial, and unclear verification. Lastly, prospective and retrospective data collection were collapsed and compared with unclear data collection according to the summary RDORs from the univariable model. Blind assessment of prediction and outcome was not clearly reported in 207 (72.1%) of the validation studies. Therefore, this feature was excluded in the multivariable model.

**Fig 3:**
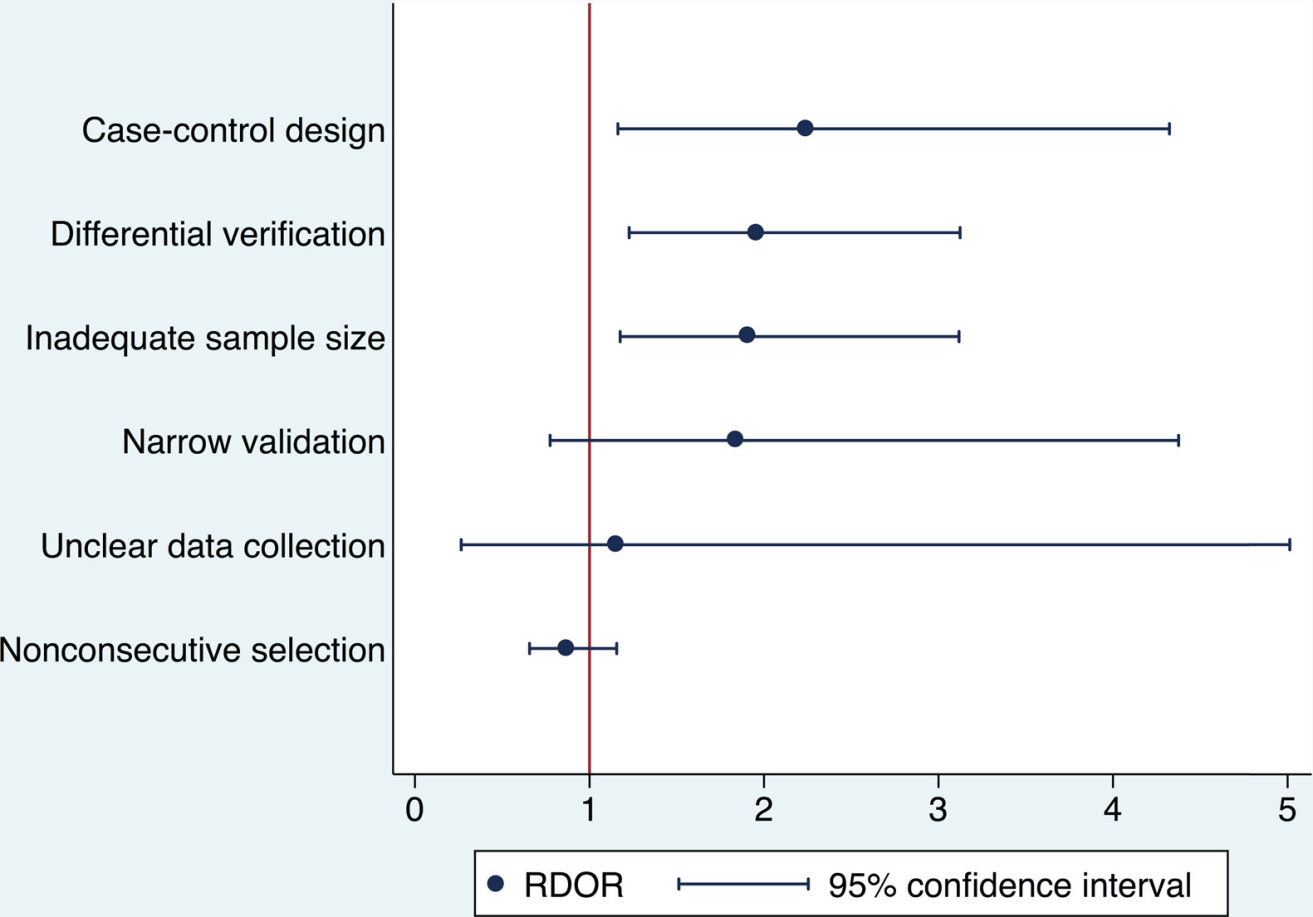
Influence of design characteristics on the performance of clinical prediction rule in multivariable analysis.

The results of the multivariable analysis are presented in Fig 3. Validation studies using case-control design produced the largest summary RDOR of 2.2 (95% CI: 1.2–4.3) compared to the validation studies with cohort design and unclear disease spectrum when controlled for the influence of other design characteristics in the multivariable model. The summary RDOR for differential verification compared to complete, partial, and unclear verification was 2.0 (95% CI: 1.2–3.1) indicating that validation studies using differential verification are likely to overestimate DOR by twofold. Validation studies conducted with inadequate sample size produced a summary RDOR of 1.9 (95% CI: 1.2–3.1).

The summary RDOR of narrow validation was 1.8 with a large 95% confidence interval of 0.8 to 4.4. When an outlier meta-analysis that produced a large coefficient in meta-regression was excluded, the summary RDOR of narrow validation was reduced to 1.3 with a less wide 95% confidence interval of 0.6 to 2.8. The outlier meta-analysis summarized performance of HADS, the Hospital Anxiety and Depression Scale, in palliative care setting [55].

Validation studies with unclear data collection methods produced a summary RDOR that was 20% larger than the summary RDOR of validation studies that clearly used prospective or retrospective data collection. However, the 95% confidence interval of the summary RDOR for validation studies with unclear data collection was 0.3 to 5.0. The wide 95% confidence interval may have been caused by the small number of heterogenous meta-analyses (*I* 2 = 65.1%) contributing to the estimation of the summary RDOR in this category.

Random-effects assumption between the results of meta-regressions was tested in sensitivity analysis. The conclusions in univariable and multivariable models were unaffected under fixed effect assumption. From 78 randomly selected validation studies, a second set of data were extracted independently for comparison. Cohen’s kappa was 0.64, 0.39, 0.05, 0.24, 0.00 and 0.26 for sample size, patient selection, spectrum, validation type, verification and data collection.

## Discussion

In this meta-epidemiological study, we investigated whether validation studies with less than optimal design characteristics are associated with the overestimation of predictive performance. We also evaluated whether studies validating clinical prediction rules are published while providing sufficient descriptions of design and reporting characteristics recommended in methodological standards.

Our results showed that many validation studies are conducted using design characteristics inconsistent with recommendations from methodological standards. The results also demonstrated that validation studies with design shortcomings overestimate the predictive performance. Among the design characteristics examined, case-control design was associated with the largest overestimation of predictive performance. This is consistent with the previous observation by Lijmer et al. [23] that using case-control design in diagnostic test accuracy studies lead to the largest overestimation of test accuracy. Case-control design was uncommon in diagnostic test accuracy studies according to the previous reports by Lijmer et al. [23] and Rutjes et al. [24] where it was used in 2.3% and 8.6% of their studies respectively. It is concerning since case-control design was used in 19.2% of validation studies of clinical prediction rule in our study. We used the definition of case-control design by Rutjes et al. [31] which included a "reversed-flow" design where an outcome is determined before the prediction is assessed. The use of this definition may have lead to the detection of validation studies using less obvious case-control design. Furthermore, the higher prevalence of case-control design in our sample may reflect lower overall methodological quality of validation studies compared to diagnostic test accuracy studies. Readers should pay close attention to detect case-control design which is relatively common among validation studies of clinical prediction rule and should use caution interpreting the results if a clinical prediction rule was validated using case-control design.

Although differential verification was less commonly observed compared to case-control design in the present study sample, it was also associated with substantial overestimation of predictive performance. This finding is similar to the previous observation by Lijmer et al. [23] in diagnostic test accuracy studies. Clinicians evaluating the performance of clinical prediction rule should be careful with the results of a validation study when subsets of predictions were verified with different reference standards.

The results showed that 87.1% of validation studies used less than sufficient number of subjects for validation of logistic regression model. These studies with inadequate sample size were associated with the overestimation of predictive performance by almost twofold. This is a confirmation of findings from simulations by Vergouwe et al. [28] and Steyerberg [11] that at least 100 subjects with the outcome and 100 subjects without the outcome were needed to detect a modest invalidity in logistic regression models.

Although it is logical to presume that clinical prediction rules would perform less well in broad validation, our study failed to observe an association between the validation type and the performance of clinical prediction rule when controlled for the influence of other design characteristics. It was common to find that all or nearly all studies included in a meta-analysis were classified as the same validation type (e.g. narrow validation) and a regression coefficient could not be generated for this feature in many meta-regressions. Meta-analyzing a small number of regression coefficients may have resulted in the imprecise estimation of a summary RDOR. Nonconsecutive selection does not appear to influence the performance of clinical prediction rule in validation studies. This finding is similar to previous observations by Lijmer et al. [23] and Stengel et al. [57].

Potential clinical implication of findings from the present study is discussed in the following example. According to one of the articles in the present study sample that validated classification criteria for rheumatoid arthritis in an outpatient setting, the sensitivity and specificity of 1987 American College of Rheumatology (ACR) criteria were both 90% [58]. However, the DOR of 81 calculated from the sensitivity and specificity may have been overestimated by 4.2 fold since this study was conducted using inadequate sample size as well as case-control design. This means that if adequate sample size and cohort design were used, the DOR would have been about 19 with corresponding sensitivity and specificity of 81%.

**Fig 4:**
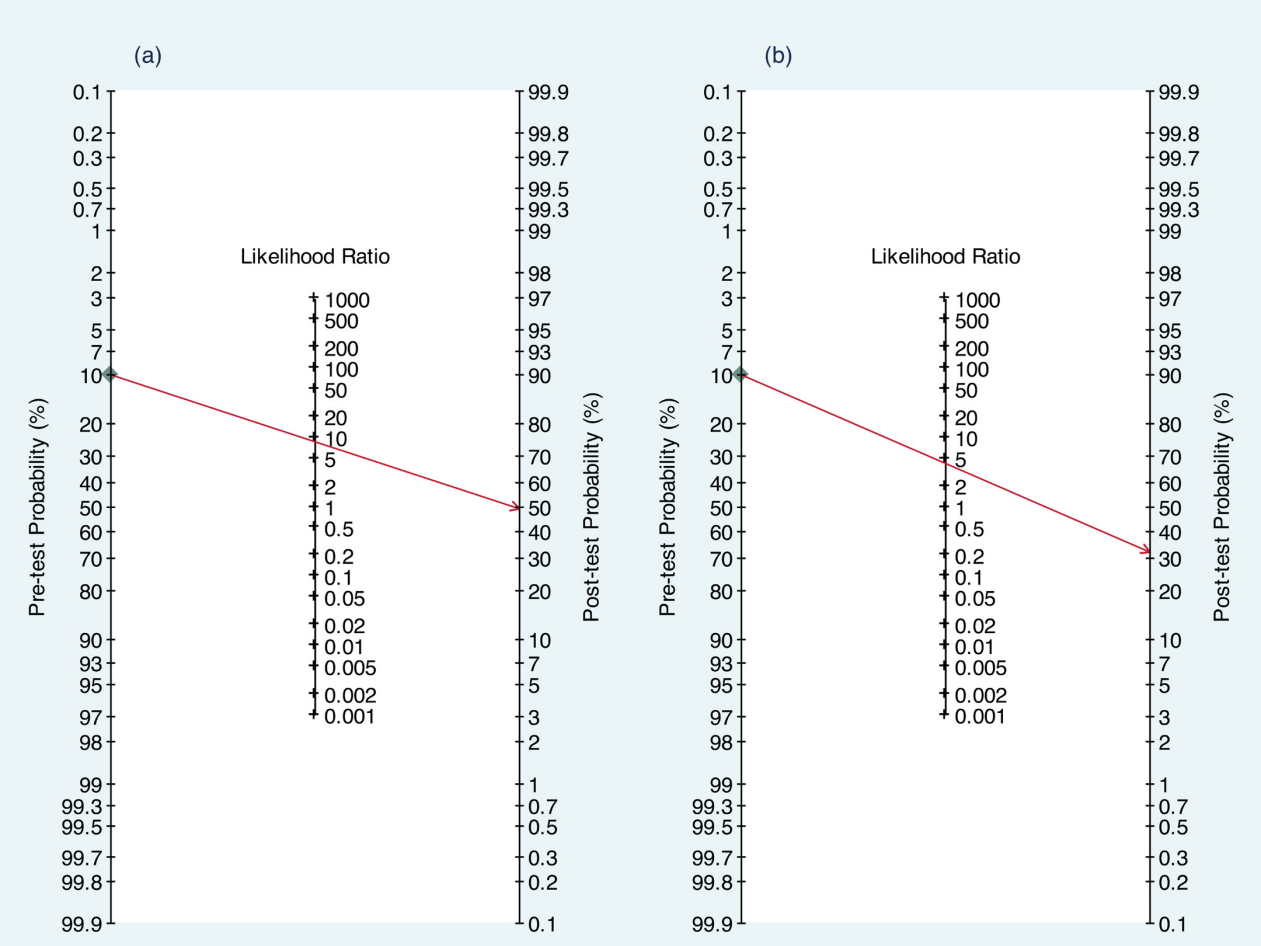
Fagan nomogram. Applying the sensitivity and specificity of (a) 90% as presented in the validation study [58] and (b) 81% from an unbiased study to a patient with 10% probability of rheumatoid arthritis.

Fig 4 illustrates potential clinical consequences of applying biased results using a hypothetical patient with 10% risk of rheumatoid arthritis. If the presence of rheumatoid arthritis is predicted with 1987 ACR criteria using the sensitivity and specificity of 90% as presented by the authors [58], the probability of rheumatoid arthritis in this patient would be 50%. On the other hand, applying the sensitivity and specificity of 81% from a hypothetical unbiased study, the probability of rheumatoid arthritis in this patient would be only 32%. In summary, applying the potentially biased results from validation studies with design shortcomings may produce overly confident clinical predictions. Consequently, unnecessary or potentially harmful therapy may be given to patients and life saving intervention may be withheld.

The reporting quality of validation studies included in the present study sample was poor. Inadequate descriptions of study site, reliability, and clinical prediction rule were highly prevalent. The results presented with confidence intervals and follow-up of study participants using flow diagram were found only in a small number of validation studies which is consistent with the findings from a recent systematic review [18]. Many validation studies did not describe whether blind assessments of clinical prediction rule and outcome were carried out.

For the findings of validation studies to be trusted and to be safely applied to clinical practice, the design, conduct and analysis of validation study must be clearly and accurately described. Unfortunately, the existing evidence including our study indicates key information is often missing or not clearly reported in validation studies. Collins et al. [59] published the TRIPOD (Transparent Reporting of multivariable prediction model for Individual Prognosis Or Diagnosis) statement which is a 22-item checklist for reporting derivation and validation of clinical prediction models. Although many methodological features evaluated in our study are included in the TRIPOD statement, there are items that are not examined in our study. Future research should examine the impact of TRIPOD statement in reporting of prediction rule studies.

The main limitation of this study is that only data from a limited number of validation studies could be independently assessed by other reviewers. Restraints in time and funding prohibited complete independent verification of data. Furthermore, there were considerable disagreements in interpretations of design characteristics between reviewers. Unfortunately, clear resolution of the disagreement was impossible for most cases due to ambiguity in description of these design characteristics in validation studies. Secondly, the small number of validation studies included in meta-analyses of clinical prediction rule studies prohibited incorporating more covariates in the multivariable model. As meta-analyses of clinical prediction rule studies become more readily available, sampling the meta-analyses with a greater number of validation studies may allow for the examination of additional covariates that influence the predictive performance. Finally, a meta-meta-analytic approach used in our study does not formally account for the clustering of DORs among clinical domains or clinical prediction rules compared to a multilevel regression modeling approach. Although this method performed well in a meta-epidemiological study of clinical trials [36], its robustness is not known in handling heterogenous validation studies of prediction rules and meta-analyses of validation studies.

In conclusion, our study demonstrated that many validation studies of clinical prediction rule are published without complying with reporting and design characteristics recommended in the methodological standards. The results of validation studies should be interpreted with caution if design shortcomings such as use of case-control design, differential verification or inadequate sample size are present as the predictive performance may have been overestimated. Conscious efforts are needed to improve the quality of design, conduct and reporting of validation studies.

## Supporting Information

S1 Fig Search strategies for systematic reviews of clinical prediction rule studies. (PDF)

S2 Fig Flow diagram. Selection of (a) systematic reviews and (b) validation studies.
(PDF)

S1 File Dataset for multivariable model. (XLSX)

S1 Table Systematic reviews of clinical prediction rule studies included in analysis. (PDF)

S2 Table Univariable analysis. Influence of design characteristics on the performance of clinical prediction rule.
(PDF)
