# Supplementary material for: Design Characteristics Influence Performance of Clinical Prediction Rules in Validation: A Meta-Epidemiological Study
Source: PLoS One. 2016 Jan 5;11(1):e0145779. doi: 10.1371/journal.pone.0145779 (PMC4701404; doi:10.1371/journal.pone.0145779)
Supplement: S1 Fig — (PDF) [file pone.0145779.s001.pdf]

S1 Appendix. Search strategies for systematic reviews of clinical prediction rule studies.

(a)

Medline (OvidSP™, 1946 to 2011)
1. predict:.mp.
2. scor:.tw.
3. observ:.mp.
4. 1 or 2 or 3
5. Cochrane database of systematic reviews.jn.
6. search.tw
7. meta-analysis.pt.
8. medline.tw.
9. systematic review.tw.
10. 5 or 6 or 7 or 8 or 9
11. 4 and 10
12. limit 11 to yr="2006 - 2010"

(b)

Embase (OvidSP™, 1974 to 2011)
1. predict:.tw.
2. exp methodology/
3. validat:.tw.
4. 1 or 2 or 3
5. meta-analysis.tw.
6. systematic review.tw.
7. MEDLINE.tw
8. 5 or 6 or 7
9. 4 and 8
10. limit 9 to yr="2006 - 2010"

(c)

Medion database
1. Systematic Reviews of Diagnostic Studies
2. limit 1 to 2006
3. limit 1 to 2007
4. limit 1 to 2008
5. limit 1 to 2009
6. limit 1 to 2010

(d)

Cochrane library
1. (predict*):ti,ab,kw
2. (rule*):ti,ab,kw
3. (decision* model*):ti,ab,kw
4. (decision* guide*):ti,ab,kw
5. (decision* tool*):ti,ab,kw
6. (decision* support*):ti,ab,kw
7. (prognos* model*):ti,ab,kw
8. (prognos* tool*):ti,ab,kw
9. (#1 or #2 or #3 or #4 or #5 or #6 or #7 or #8) from 2006 to 2010 in Cochrane Reviews and Other Reviews
