# Supplementary figures and images for: Design Characteristics Influence Performance of Clinical Prediction Rules in Validation: A Meta-Epidemiological Study
Source: PLoS One. 2016 Jan 5;11(1):e0145779. doi: 10.1371/journal.pone.0145779 (PMC4701404; doi:10.1371/journal.pone.0145779)

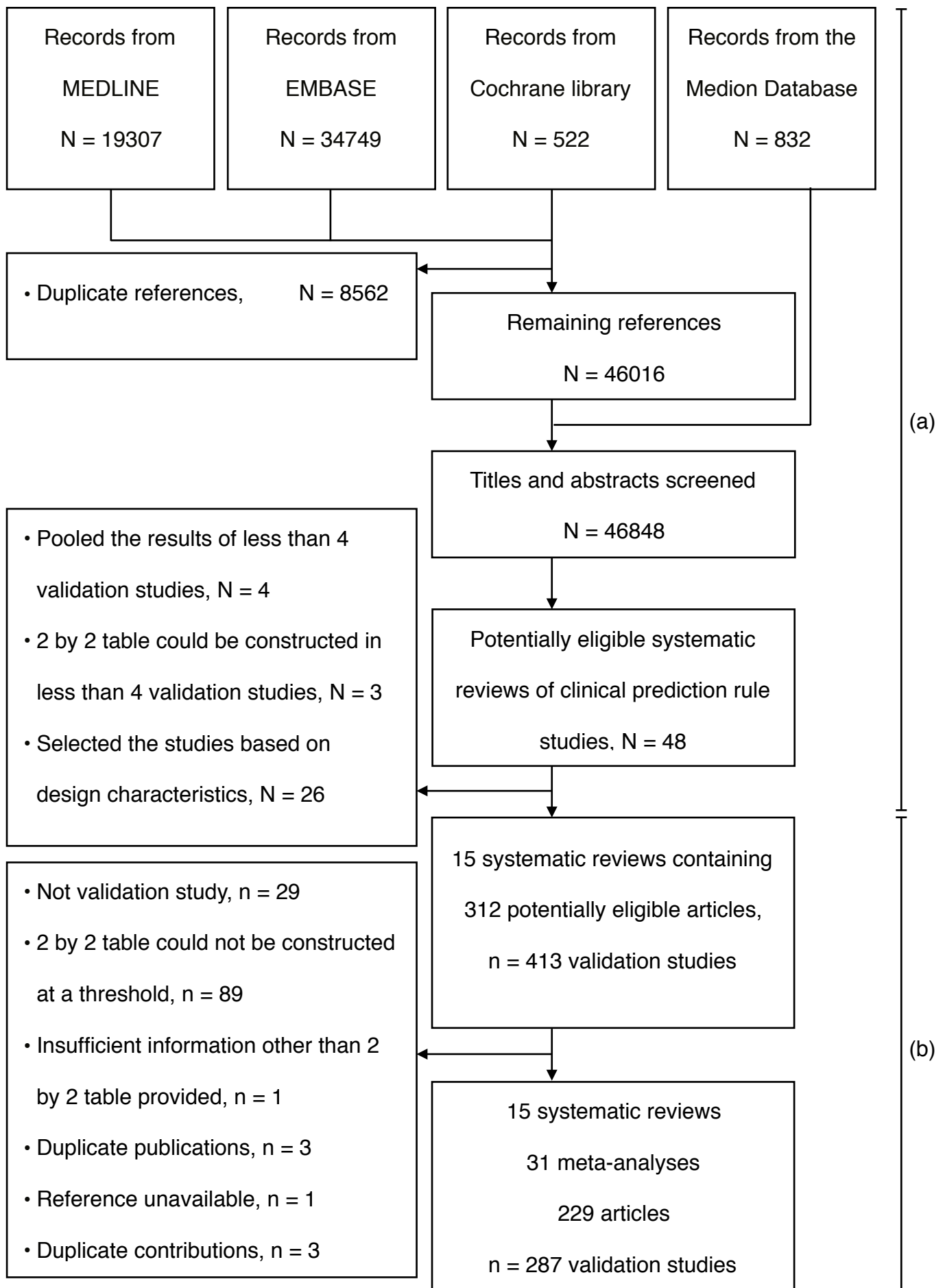

**S2 Appendix. Flow diagram.** Selection of (a) systematic reviews and (b) validation studies.

Supplement: S2 Fig — Selection of (a) systematic reviews and (b) validation studies. (PDF) [file pone.0145779.s002.pdf]
