# Supplementary material for: Design Characteristics Influence Performance of Clinical Prediction Rules in Validation: A Meta-Epidemiological Study
Source: PLoS One. 2016 Jan 5;11(1):e0145779. doi: 10.1371/journal.pone.0145779 (PMC4701404; doi:10.1371/journal.pone.0145779)
Supplement: S1 Table — (PDF) [file pone.0145779.s004.pdf]

S3 Appendix. Systematic reviews of clinical prediction rule studies included in the study.

Systematic review	Clinical prediction rule	Outcome	Number of Validation studies
**Banal et al., 2009**	American College of Rheumatology 1987 Criteria	Early rheumatoid arthritis determined by expert opinion	6
	American College of Rheumatology 1987 Criteria	Established rheumatoid arthritis determined by expert opinion	10
**Brennan et al., 2010**	Hospital Anxiety and Depression Scale (HADS-D)	Major depression determined by standardized interview	8
	Hospital Anxiety and Depression Scale (HADS-D)	All depressive disorders determined by standardized interview	10
**Ceriani et al., 2010**	3-level Wells score	Pulmonary embolism define by negative D-dimer with intermediate or low clinical probability, ventilation-perfusion lung scan, helical computed tomography or pulmonary angiography	13
	2-level Wells score	Pulmonary embolism define by negative D-dimer with intermediate or low clinical probability, ventilation-perfusion lung scan, helical computed tomography or pulmonary angiography	6
	Geneva score	Pulmonary embolism define by negative D-dimer with intermediate or low clinical probability, ventilation-perfusion lung scan, helical computed tomography or pulmonary angiography	5
**Dowling et al., 2009**	Ottawa Ankle Rules	Ankle or mid-foot fracture in children determined by X-ray or ankle, foot or proxy measure such as telephone follow up	10
**Geomini et al., 2009**	Sassone model	Ovarian malignancy based on histology	17
	Lerner model	Ovarian malignancy based on histology	7
	DePriest model	Ovarian malignancy based on histology	7
	Ferrazi model	Ovarian malignancy based on histology	6
	Risk of Malignancy Index 1	Ovarian malignancy based on histology	12
	Risk of Malignancy Index 2	Ovarian malignancy based on histology	6

Systematic review	Clinical prediction rule	Outcome	Number of Validation studies
**Gilbody et al., 2007**	Patient Health Questionnaire (PHQ) 9	Depression determined by standardized diagnostic interview	9
**Giles and Rothwell, 2010**	ABCD score	Stroke based on World Health Organization (WHO) Criteria	8
	ABCD2 score	Stroke based on World Health Organization (WHO) Criteria	6
**Harrington et al., 2010**	St. Thomas's risk assessment tool (STRATIFY)	Fall in hospitalized patients	10
**Hewitt et al., 2009**	Edinburgh Postnatal Depression Scale (EPDS)	Major depression determined by standardized interview	16
	Edinburgh Postnatal Depression Scale (EPDS)	Major and minor depression determined by standardized interview	16
	Edinburgh Postnatal Depression Scale (EPDS)	Any psychiatric disorder determined by standardized interview	5
**McPhail et al., 2010**	Kings's College Hospital Criteria (KCC)	Emergency liver transplantation or death	11
**Mitchell et al., 2010b**	Geriatric Depression Scale (GDS) 30	Depression in medical settings and nursing homes determined by structured interview	21
	Geriatric Depression Scale (GDS) 15	Depression in medical settings and nursing homes determined by structured interview	14
**Mitchell et al., 20101a**	Geriatric Depression Scale (GDS) 30	Depression in primary care determined by structured interview	7
	Geriatric Depression Scale (GDS) 15	Depression in primary care determined by structured interview	8
**Mitchell et al., 2010c**	Hospital Anxiety and Depression Scale (HADS-D)	Depression determined by structured interview	7
	Hospital Anxiety and Depression Scale (HADS-T)	Depression determined by structured interview	5
	Hospital Anxiety and Depression Scale (HADS-T)	Any mental illness determined by structured interview	4
**Serrano et al., 2010**	San Francisco Syncope Rule (SFSR)	Adverse outcome after stroke (death, MI, arrhythmia, PE, stroke, subarachnoid hemorrhage, hemorrhage, ED return visit and hospitalization)	7
**Warnick et al., 2008**	Child Behavior Checklist (CBCL)	Any psychiatric disorder determined by structured interview	10
