# Supplementary material for: Design Characteristics Influence Performance of Clinical Prediction Rules in Validation: A Meta-Epidemiological Study
Source: PLoS One. 2016 Jan 5;11(1):e0145779. doi: 10.1371/journal.pone.0145779 (PMC4701404; doi:10.1371/journal.pone.0145779)
Supplement: S2 Table — Influence of design characteristics on the performance of clinical prediction rule. (PDF) [file pone.0145779.s005.pdf]

S4 Appendix. Univariable analysis. Influence of design characteristics on the performance of clinical prediction rule.

Design property	Level	Study	(%)	RDOS (95% CI)	I^2%
Sample size	Adequate	37	(12.9)	1	
	Inadequate	250	(87.1)	1.5 (1.2 - 1.9)	13.2
Patient selection	Consecutive	131	(45.6)	1	
	Nonconsecutive	45	(15.7)	1.3 (0.9 - 1.9)	0
	Unclear	111	(38.7)	1.1 (0.9 - 1.5)	38.5
Disease spectrum	Cohort	122	(42.5)	1	
	Case-control	55	(19.2)	1.3 (0.6 - 2.5)	85.3
	Unclear	110	(38.3)	0.6 (0.4 - 1.0)	66.1
Validation type	Broad	52	(18.1)	1	
	Narrow	205	(71.4)	1.8 (0.8 - 3.8)	62.0
	Unclear	30	(10.5)	3.1 (0.9 - 10.5)	35.2
Assessment	Blind	72	(25.1)	1	
	Non-blind	8	(2.8)	1.0 (0.7 - 1.6)	0
	Unclear	207	(72.1)	1.2 (0.8 - 1.7)	39.3
Verification	Complete	145	(50.5)	1	
	Partial	27	(9.4)	0.9 (0.5 - 1.5)	57.3
	Differential	51	(17.8)	1.8 (1.1 - 3.1)	
	Unclear	64	(22.3)	0.7 (0.4 - 1.2)	54
Data collection	Prospective	210	(73.2)	1	
	Retrospective	62	(21.6)	0.9 (0.6 - 1.4)	41.9
	Unclear	15	(5.2)	1.4 (0.8 - 2.5)	1
